# *AHK3*-Mediated Cytokinin Signaling Is Required for the Delayed Leaf Senescence Induced by *SSPP*

**DOI:** 10.3390/ijms20082043

**Published:** 2019-04-25

**Authors:** Yanan Wang, Xiyu Zhang, Yanjiao Cui, Lei Li, Dan Wang, Yuanyuan Mei, Ning Ning Wang

**Affiliations:** Tianjin Key Laboratory of Protein Sciences, Department of Plant Biology and Ecology, College of Life Sciences, Nankai University, Tianjin 300071, China; wyanan0306@163.com (Y.W.); zhangxiyunku@163.com (X.Z.); cuiyj189@163.com (Y.C.); lei.li@nankai.edu.cn (L.L.); wangdan629@nankai.edu.cn (D.W.)

**Keywords:** cytokinin signaling, AHK3, leaf senescence, SSPP

## Abstract

Leaf senescence is a highly-programmed developmental process regulated by an array of multiple signaling pathways. Our group previously reported that overexpression of the protein phosphatase-encoding gene *SSPP* led to delayed leaf senescence and significantly enhanced cytokinin responses. However, it is still unclear how the delayed leaf senescence phenotype is associated with the enhanced cytokinin responses. In this study, we introduced a cytokinin receptor *AHK3* knockout into the *35S:SSPP* background. The phenotypic analysis of double mutant revealed that *AHK3* loss-of-function reversed the delayed leaf senescence induced by *SSPP*. Moreover, we found the hypersensitivity of *35S:SSPP* to exogenous cytokinin treatment disappeared due to the introduction of *AHK3* knockout. Collectively, our results demonstrated that *AHK3*-mediated cytokinin signaling is required for the delayed leaf senescence caused by *SSPP* overexpression and the detailed mechanism remains to be further elucidated.

## 1. Introduction

Senescence represents the final stage of leaf development, featured by degradation and recycling of nutrients in senescent leaves to actively growing organs [1]. It is not only critical for a plant’s fitness and nutrient relocation, but also crucial for crop yield and quality in agricultural perspectives [2]. As a process highly regulated by genetic programing, both the initiation and progression of leaf senescence occur in response to environmental factors and endogenous signals such as plant hormones [3]. As one of the ancient plant hormones [4], cytokinin has long been widely acknowledged as a negative regulator of leaf senescence [5]. In *Arabidopsis*, cytokinin is perceived by three different receptors, namely AHK2, AHK3, and AHK4/CRE1/WOL [6,7,8]. Among the three receptors, AHK3 plays a major role in leaf senescence regulation through a specific phosphorylation of the B-type response regulator ARR2 [9]. Cytokinin-mediated leaf longevity also involves the cytokinin response factors [10,11], and the downstream extracellular invertase that may function in source-to-sink nutrient mobilization [12].

We previously found that overexpression of a PP2C type protein phosphatase-encoding gene *SSPP* led to significantly delayed leaf senescence and enhanced cytokinin responses in *Arabidopsis* [13]. However, it remains largely unknown how *SSPP*-mediated delayed leaf senescence signaling is associated with cytokinin signaling. Therefore, we obtained *ahk3-3/35S:SSPP* hybrid *Arabidopsis*, and found that knockout of *AHK3* reversed the delayed leaf senescence caused by *SSPP* overexpression. The hypersensitivity of *35S:SSPP* to exogenous cytokinin treatment is lost in *ahk3-3/35S:SSPP*. These results together indicate that *AHK3*-mediated cytokinin signaling is required for the delayed leaf senescence induced by *SSPP* overexpression.

## 2. Results and Discussion

**Figure 1:**
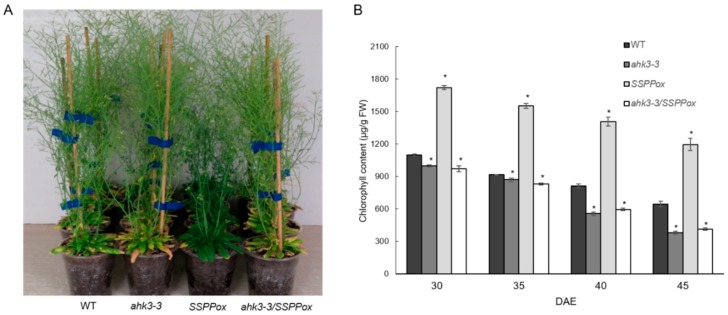
Loss of *AHK3* reversed the delayed leaf senescence mediated by *SSPP.* (**A**) The 59-day-old *ahk3-3*, *35S:SSPP*(*SSPPox*), *ahk3-3/SSPPox* and wild-type (WT) control *Arabidopsis* were photographed. (**B**) chlorophyll contents in the sixth leaf of *ahk3-3*, *SSPPox*, *ahk3-3/SSPPox*, and wild-type control *Arabidopsis* were determined at 30, 35, 40 and 45 days after emergence (DAE). FW, fresh weight. Three biological replicates with at least three technical repeats were done. Error bars represent standard errors (SE). Asterisks indicate significant differences with the wild-type at each time point (α = 0.05).

In order to gain a better understanding of the molecular mechanism underlying *SSPP*-mediated delayed leaf senescence, we generated hybrid *Arabidopsis* by introducing cytokinin receptor *AHK3* knockout [14] into *35S:SSPP* background(*SSPPox*). The genotyping of the hybrid *Arabidopsis* was confirmed by semi-quantitative RT-PCR using the *Tip41-like* gene as an internal control (Figure S1). Phenotypic analysis assay was performed with reciprocal comparisons of *ahk3-3/SSPPox*, *SSPPox*, *ahk3-3* mutants and the wild-type (WT) control (Figure 1). In line with our previous report [13], the leaves of *SSPP* overexpressing *Arabidopsis* exhibited significantly delayed senescence in comparison to the ones of the WT. Conversely, the leaves of the *ahk3-3* mutant showed precocious senescence. Of particular note, the hybrid *Arabidopsis ahk3-3/SSPPox* displayed accelerated leaf senescence which was similar to the *ahk3-3* mutant but reversed the late senescence of *SSPPox* at 59 days after emergence (DAE) (Figure 1A). We also compared the rosette diameter, plant height, bolting time, and flowering period of all the different plants (Figure S2). Compared to the WT, *SSPPox* showed smaller rosettes, reduced plant height, delayed bolting time as well as flowering time, which is consistent with our previous findings [13]. However, no differences were observed among *ahk3-3* and *ahk3-3/SSPPox*.

To get a quantitative comparison in leaf senescence, we measured the chlorophyll contents in the sixth leaf of all the above-mentioned transgenic plants and WT from 30 to 45 DAE. The results showed that contents of chlorophyll were significantly increased in the leaves of *SSPPox* while they were declined in the leaves of the *ahk3-3* mutant at all stages in comparison to those in the WT (Figure 1B). In the leaves of *ahk3-3/SSPP-ox*, chlorophyll contents were lower than in WT and *SSPPox*, but were at similar levels to those in the *ahk3-3* mutant (Figure 1B). This demonstrates that the hybrid *Arabidopsis* showed similar senescence phenotype to the *ahk3-3* mutant.

**Figure 2:**
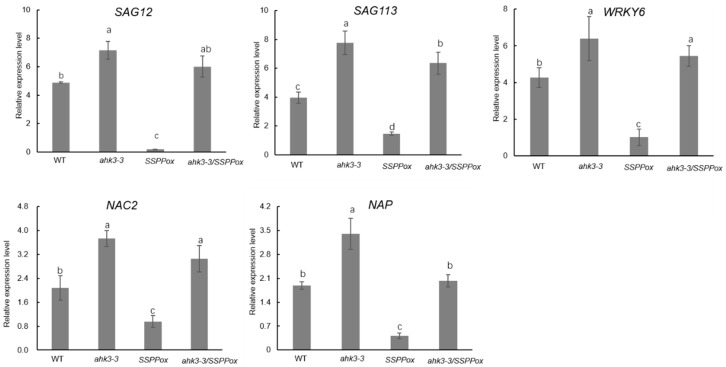
Knockout of *AHK3* increased the transcripts levels of several critical senescence-associated marker genes in *35S:SSPP* transgenic *Arabidopsis.* The relative expression levels of several senescence-associated marker genes were determined in *ahk3-3*, *SSPPox*, *ahk3-3/SSPPox*, and wild-type control *Arabidopsis.* RNA was extracted from the fifth and sixth leaves of 28-day-old plants and quantitative RT-PCR was performed as described in materials and methods using the *TIP41-like* gene as an internal control. Three biological replicates with at least three technical repeats were done. Different letters indicate statistically significant differences based on analysis of variance (ANOVA) (α = 0.05). Error bars represent standard errors (SE).

Consistent with chlorophyll contents, expressions of senescence-associated marker genes including *SAG12* [15], *SAG113* [16], *NAP* [17], *WRKY6* [18], and *NAC2* [19] were remarkably inhibited in the leaves of *SSPPox* but were significantly enhanced in the leaves of *ahk3-3* mutant and the hybrid *Arabidopsis ahk3-3/SSPPox* (Figure 2). These results indicated that knockout of *AHK3* reversed the delayed leaf senescence in *SSPP* overexpressing *Arabidopsis*.

**Figure 3:**
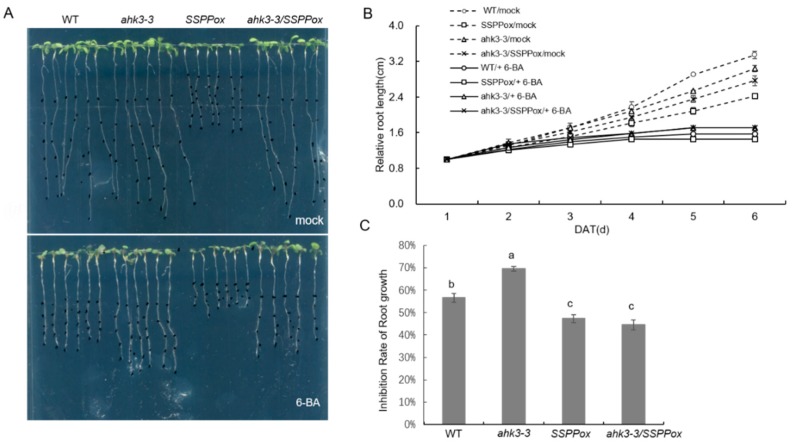
Loss of *AHK3* diminished the hypersensitivity of *SSPP*-overexpressing *Arabidopsis* to exogenous cytokinin treatment. (**A**) Four-day-old WT, *ahk3-3*, *SSPPox*, and *ahk3-3/SSPPox* transgenic seedlings were grown on vertical plates containing either 6 μM 6-BA or a mock solution for additional 6 days and photographed. (**B**) Comparison of relative root length of WT, *ahk3-3, SSPPox*, and *ahk3-3/SSPPox* transgenic seedlings under either mock or 6-BA treatment every day for a total period of six days. (**C**) Comparison of growth inhibition rates of main root growth of WT, *ahk3-3, SSPPox*, and *ahk3-3/SSPPox* at 6 days after treatment (DAT). Data shown are the typical results of three biological replicates. Different letters indicate statistically significant differences based on analysis of variance (ANOVA) (α = 0.05). Error bars represent SE.

To test the sensitivity of various *Arabidopsis* lines to cytokinin, we treated them with exogenous cytokinin (6-benzyladenine, 6-BA). Four-day-old seedlings of *SSPPox*, *ahk3-3*, *ahk3-3/SSPPox*, and WT were transferred to vertical half-strength MS plates containing either mock solution or 5 μM 6-BA and kept growing for another six days. Upon mock treatment, the *SSPPox* seedlings showed retarded root growth compared to the WT as reported previously [13]. By contrast, the root development of the *ahk3-3* mutant and hybrid *Arabidopsis ahk3-3/SSPPox* did not show any significant difference with WT (Figure 3A,B). Under 6-BA treatment, *SSPP* overexpressing seedlings exhibited significantly inhibited root growth compared with WT (Figure 3A,B). As expected, the hybrid *Arabidopsis ahk3-3/SSPP-ox* displayed reduced sensitivity to 6-BA application similar to the *ahk3-3* mutant (Figure 3A,B). We also compared the inhibitory rates of root growth in all the above-mentioned transgenic seedlings and WT at six days after 6-BA treatment compared to the mock treatment. It was found that the inhibitory rate was significantly higher in *SSPPox* while was remarkably reduced in both the *ahk3-3* mutant and the hybrid *Arabidopsis ahk3-3/SSPPox* in comparison to that in WT (Figure 3C). These results indicated that hypersensitivity of *SSPP* overexpressing *Arabidopsis* to exogenous cytokinin application is linked with *AHK3*. Taken together, the *SSPP*-induced delayed leaf senescence and enhanced cytokinin responses are closely linked, and its hypersensitivity to cytokinin is associated with *AHK3*-mediated cytokinin signaling.

The essential role of *AHK3* as the major contributor in regulating leaf longevity was firstly reported by Kim et al. [9] based on the senescence analysis assays of dark-induced excised leaves. But this study claimed that the loss-of-function mutant *ahk3* did not show a significantly early senescence phenotype during age-dependent senescence. Conversely, a recent study from Danilova et al. [20] argued that the *ahk3* single mutant displayed a slightly accelerated vegetative growth. In this study, we carried out a thorough phenotypic analysis, and compared the chlorophyll contents as well as the transcript levels of several senescence-associated marker genes (*SAGs*) in the *ahk3-3* mutant and the wild-type control plants. From these integrated analyses, we found the *ahk3-3* mutant not only showed declined chlorophyll contents (Figure 1B) but also significantly up-regulated expressions of five critical *SAGs* such as *SAG12*, *SAG113*, *NAP*, *WRKY6*, and *NAC2* (Figure 2), which collectively demonstrate that loss of *AHK3* leads to accelerated leaf senescence.

The molecular mechanisms underlying the cross-talk between *SSPP*-mediated delayed leaf senescence signaling and *AHK3*-mediated cytokinin responses will be a subject for further exploration. Upon the binding of cytokinin, AHK3 was reported to phosphorylate the downstream phosphortransmitter proteins, which are then translocated to the nucleus and phosphorylate specifically the type-B response regulator ARR2. ARR2 then activates the expressions of downstream target genes and induce a range of physiological processes and cellular responses in the cytoplasm and the organelle during dark-induced leaf senescence [9]. However, the mechanism underlying the role of AHK3 during natural leaf senescence needs to be further studied. Recent cell biological and biochemical evidences demonstrated that AHK3 was localized in the endoplasmic reticulum (ER) [21]. Our previous research found that the protein phosphatase SSPP was localized in the cytoplasm [13]. Moreover, *SSPP* overexpression resulted in increased expressions of several cytokinin-responsive markers genes such as type-A response regulators *ARR5* and *ARR6*. In addition, the *SSPP* loss-of-function mutant named *sspp-1* exhibited no significant differences in growth and development when compared to wild-type *Arabidopsis*. As this study demonstrated that *AHK3*- mediated cytokinin response is required in *SSPP*-induced delayed leaf senescence, it would be interesting to further investigate whether SSPP could directly interact with or dephosphorylate AHK3, or whether SSPP can interact with other components of the cytokinin signal transduction pathway mediated by AHK3. In addition, our group found that SSPP negatively regulates leaf senescence by directly interacting with and suppressing the leucine-rich repeat receptor-like protein kinase (LRR-RLK) SARK [13], which works as a positive regulator of leaf senescence [22]. Transcript analysis of *SARK*-overexpressing plants revealed a wide range of changes in phytohormone synthesis and signaling including a strong repression of cytokinin functions [22]. Therefore, it would be also interesting to investigate whether there are cross-talks between AHK3 and SARK, and whether the dephosphorylation of SSPP is SARK-specific. The current study laid foundation for further understanding towards the role of cytokinin in leaf senescence and the detailed signal transduction pathways of the delayed leaf senescence process mediated by *SSPP* overexpression.

## 3. Materials and Methods

### 3.1. Plant Material and Growth Condition

*Arabidopsis thaliana* (Columbia-0 ecotype) was used in this study. The loss-of-function *ahk3-3* mutant seeds [14] were provided by Professor Shuhua Yang from China Agricultural University. Seeds were surface sterilized in 10% (*v/v*) sodium hypochlorite (Tianjin Chemicals, 559, Tianjin, China) for 2 min, washed at least 10 times with sterilized water, and germinated on one half-strength Murashige Skoog (MS) medium (Duchefa Biochemie, M0222, Haarlem, the Netherlands) containing 0.8% (*w/v*) agar (Solarbio, A8190, Beijing, China), pH5.7, 1% (*w/v*) Suc (Jiangtian Chemicals, 11411, Nantong, China), supplemented with or without antibiotics, stratified at 4 °C for 2 days in the dark, and then grown in plant growth chamber at 22/19 °C with cycles of 16 h light and 8 h darkness under 100 to 150 μmol m^−2^·s^−1^ light intensity. The 10-day-old seedlings were then transferred to soil and grown under the same conditions for further experiments.

### 3.2. Generation of Hybrid Arabidopsis

The hybrid *Arabidopsis ahk3-3/SSPPox* was generated by crossing *35S:SSPP* with the *ahk3-3* mutant, which served as the male parent and female parent, respectively. Homozygous plants were identified by segregation analysis and PCR-based genotyping among the F3 progeny. All the primers used were listed in Table S1.

### 3.3. Measurements of Chlorophyll Contents

The chlorophyll contents in mesophyll cells were spectrophotometrically measured as described in Arnon (1949) [23].

### 3.4. RNA Extraction and RT-PCR Analysis of Gene Expression

RNA extraction, cDNA synthesis, and RT-PCR analysis were performed as described previously in Liu et al. [24]. The real-time RT-PCR analyses were performed on an iQ5 (Bio-Rad, Hercules, CA, USA) machine using a SYBR Green reagent (Takara, Berkeley, CA, USA) with gene-specific primers (Table S1). The relative expression levels were calculated as described previously [25]. At least three independent replicates were performed to give typical results shown here.

### 3.5. Cytokinin Response Assay

Five-day-old *35S:SSPP*(*SSPPox*), *ahk3-3*, and *ahk3-3/SSPPox* transgenic *Arabidopsis* seedlings and the wild-type control were transferred from basal half-strength MS medium to fresh induction plates containing 5 μM 6-BA (6-Benzylaminopurine, Sigma, B3408, Darmstadt, Germany) or a mock solution. During an additional growth period of indicated durations, the root lengths were measured using magnified images via ImageJ software (National Institutes of Health, Bethesda, Maryland, USA, https://imagej.nih.gov/ij/download.html). Each treatment was replicated three times.

## Supplementary Materials

The following are available online at https://www.mdpi.com/1422-0067/20/8/2043/s1, Figure S1: The genotyping of the hybrid *Arabidopsis ahk3-3/SSPPox* was confirmed by semi-quantitative RT-PCR. Figure S2: Reciprocal comparisons between *ahk3-3/SSPPox*, *ahk3-3*, *SSPPox*, and WT on rosette diameters, plant height, bolting time, and flowering time. Table S1: Primers used in this study.

## Abbreviations

DAE	Days After Emergence
DAT	Days After Treatment
LRR-RLK	Leucine-Rich Repeat Receptor-Like Protein Kinase
