# Supplementary material for: AHK3-Mediated Cytokinin Signaling Is Required for the Delayed Leaf Senescence Induced by SSPP
Source: Int J Mol Sci. 2019 Apr 25;20(8):2043. doi: 10.3390/ijms20082043 (PMC6514669; doi:10.3390/ijms20082043)
Supplement: Supplementary file 1 [file ijms-20-02043-s001.zip › Supplemental Table 1.docx]

**Supplementary Table S1**: Primers used in this study

| Primer names | Sequences (5'-3') |
| --- | --- |
| *ahk-3 F* | *CTTGTGATTGCGTTACTTGTTGCAC* |
| *ahk-3 R* | *GCAGGCCTATGGTCCACAACCACAG* |
| *ahk3-3 LB* | *TGGTTCACGTAGTGGGCCATCG* |
| *35S-F-XL* | *CGTAAAGACTGGCGAACAGTTC* |
| *kh-SSPP-2* | *GCATCACCTATGGATCTTGTGACC* |
| *NOS-1* | *ATAATCATCGCAAGACCGGCAACAG* |
| *semi-SSPP-1* | *GGCCATGGAGGTCCAGAGGCT* |
| *semi-SSPP-2* | *TCTTGTGGGCTAAGCCGCGT* |
| *seemi-AHK3-F* | *GGAGGTGCGGTTTGATTTG* |
| *semi-AHK3-R* | *GGCTGGTTGTTGTCATTCTTTC* |
| *rtAtNAP-1* | *TTACATGGGACCCGTCTCTC* |
| *rtAtNAP-2* | *CCGAACCAACTAGACTCCGA* |
| *rtNAC1-1* | *ACATCCCAAAAATGGCATGC* |
| *rtNAC1-2* | *TGCTCGGTTAGTTCTCAGCC* |
| *rtNAC2-1* | *CTTTCATTCGGGGTAGTCCA* |
| *rtNAC2-2* | *AACATAGCTCTTGTCGCCGT* |
| *rtWRKY6-1* | *CAGTTCTCTGGTGGCTCTCC* |
| *rtWRKY6-2* | *GTCAGCTGTGAGTGCCGTTA* |
| *TIP-F* | *GAAATTCAGGAGCAAGCCGTCTCAG* |
| *TIP-R* | *ATCAACTCTCAGCCAAAATCGCAAG* |
| *semi-TIP-F* | *GAAATTCAGGAGCAAGCCGTCTCAG* |
| semi-TIP-R | *ATCAACTCTCAGCCAAAATCGCAAG* |
