# Supplementary figures and images for: AHK3-Mediated Cytokinin Signaling Is Required for the Delayed Leaf Senescence Induced by SSPP
Source: Int J Mol Sci. 2019 Apr 25;20(8):2043. doi: 10.3390/ijms20082043 (PMC6514669; doi:10.3390/ijms20082043)

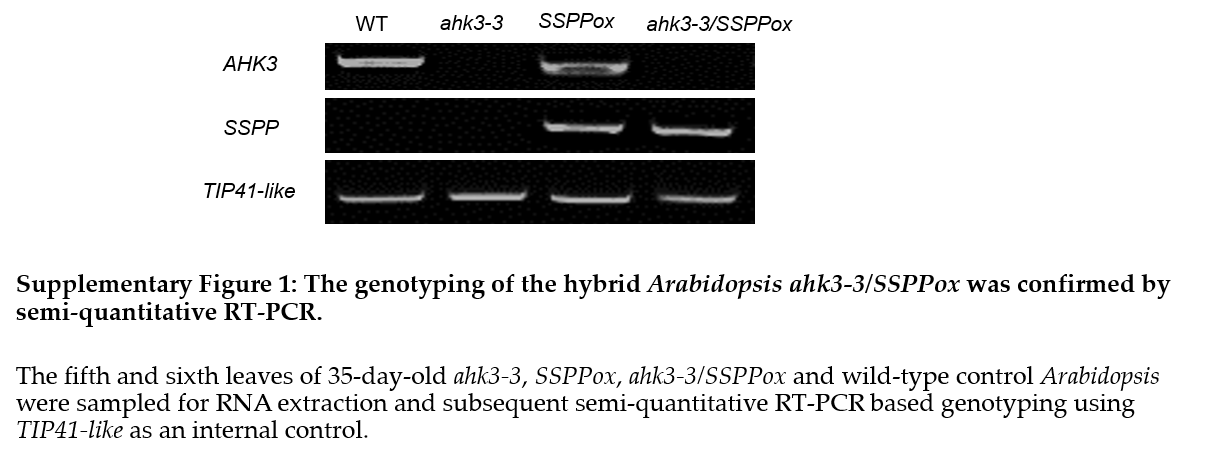

Supplement: Supplementary file 1 [file ijms-20-02043-s001.zip › Supplemental Figure 1.tif]

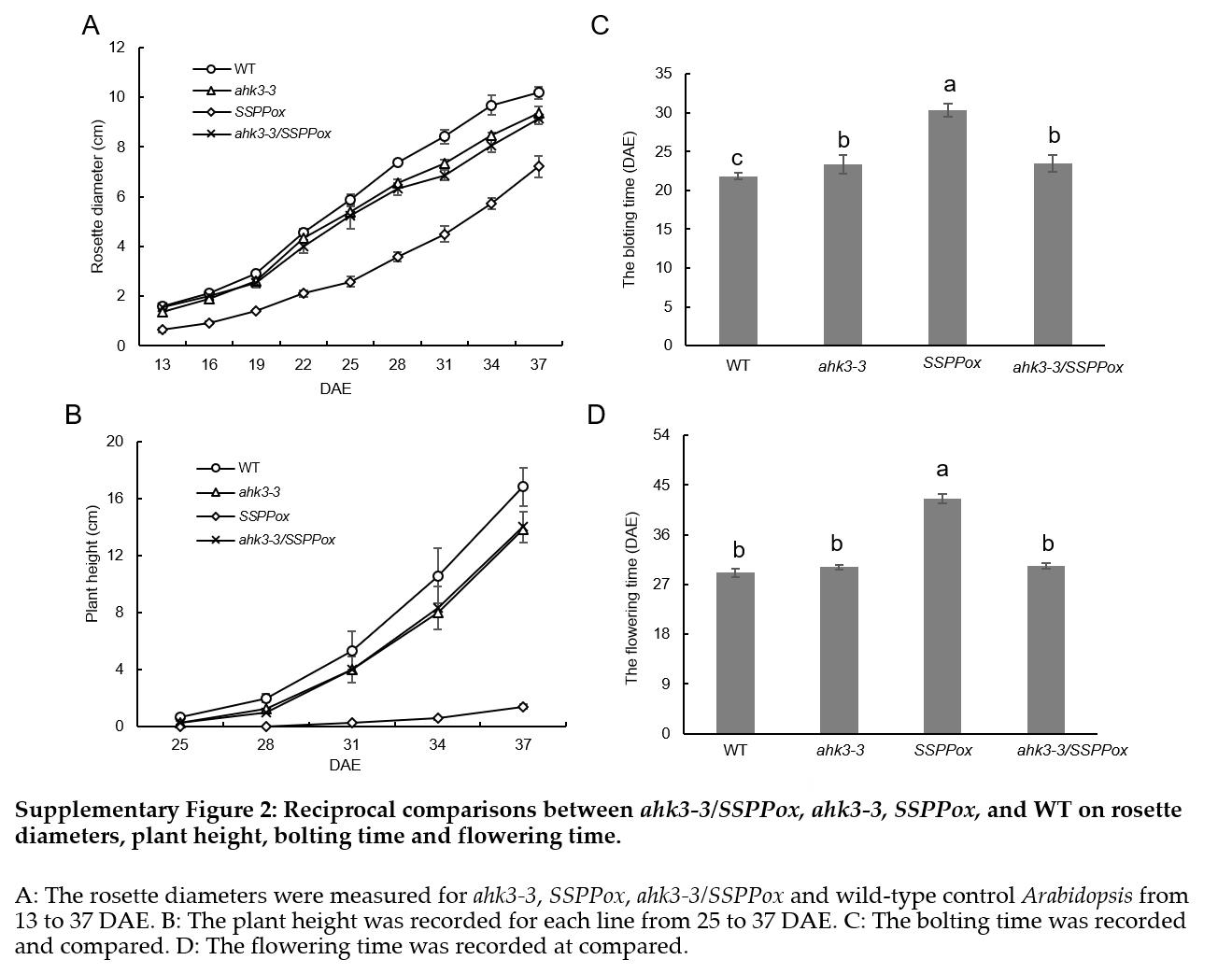

Supplement: Supplementary file 1 [file ijms-20-02043-s001.zip › Supplemental Figure 2.tif]
